# Adult Onset Vitiligo: Multivariate Analysis Suggests the Need for a Thyroid Screening

**DOI:** 10.1155/2016/8065765

**Published:** 2016-09-22

**Authors:** L. Lazzeri, R. Colucci, A. Cammi, F. Dragoni, S. Moretti

**Affiliations:** Department of Surgery and Translational Medicine, Section of Dermatology, University of Florence, Florence, Italy

## Abstract

*Background*. There are limited epidemiological studies evaluating the effect of age at onset on disease features in vitiligo.* Objectives*. To identify factors associated with adult onset vitiligo in comparison with childhood onset vitiligo.* Patients and Methods*. We retrospectively collected medical records of 191 patients. Such records included clinical examination, personal and familial medical history, laboratory evaluations, concomitant vitiligo treatment and drug assumption.* Results*. 123 patients with a disease onset after the age of 40 (adult onset vitiligo) were compared with 68 patients who developed vitiligo before the age of 12 (childhood onset vitiligo). Multivariate analysis revealed that personal history of thyroid diseases (*P* = 0.04; OR 0.4), stress at onset (*P* = 0.002; OR = 0.34), personal history of autoimmune thyroid disease (ATD) (*P* = 0.003; OR = 0.23), and thyroid nodules (*P* = 0.001; OR 0.90) were independently associated with adult onset vitiligo, whereas family history of dermatological diseases (*P* = 0.003; OR = 2.87) and Koebner phenomenon (*P* < 0.001; OR = 4.73) with childhood onset vitiligo. Moreover, in the adult onset group, concomitant thyroid disease preceded vitiligo in a statistically significant number of patients (*P* = 0.014).* Conclusions*. Childhood onset and adult onset vitiligo have different clinical features. In particular, ATD and thyroid nodules were significantly associated with adult onset vitiligo, suggesting that a thyroid screening should be recommended in this group of patients.

## 1. Introduction

Vitiligo is a chronic acquired pigmentary skin disorder, clinically characterized by well-defined asymptomatic white macules, which are the result of a loss of functional melanocytes in the epidermis. It is considered the most common pigmentary disorder, affecting 0.1–2% of the world population, with no sexual or racial preference [1].

Vitiligo can appear at any age, but it has been most frequently observed in the first two decades [2].

In particular, it tends to appear before the age of 10 in about 25% of the patients [3, 4]. However, a disease onset during adulthood is a common condition as well [5], even if to date only few studies [6, 7] have described the clinic profile of adult onset vitiligo.

We herein present the results of a retrospective observational study comparing the clinical characteristics of adult and childhood onset vitiligo through univariate analysis and multivariate conditional logistic regression.

## 2. Patients and Methods

We retrospectively collected medical records of 500 consecutive patients diagnosed with vitiligo attending the Florence University specialized vitiligo outpatient service between June 2010 and June 2013. Patients with vitiligo arisen between the ages of 12 and 40 years were excluded from the study. The remaining 191 patients were divided into two groups, according to the age at onset of the disease: the adult onset group including 123 patients with a disease onset after the age of 40 and the childhood onset group including 68 patients, both children and adults, having a disease onset before the age of 12 years.

The study design was conducted according to the Declaration of Helsinki principles and informed consent was obtained from each patient.

### 2.1. Medical Assessment

Medical records included clinical examination, personal and familial medical history, laboratory evaluations, and concomitant vitiligo treatment and drug assumption.

Medical assessment was performed using a modified VETF form [8, 9]. This evaluation individually assesses four body regions (head and neck, trunk, upper extremities, and lower extremities), examined both under natural light and under Wood lamp, for the extent of depigmentation (affected body surface percentage scored 0–100%), stage of disease (staging), scored 0–4 (normal pigmentation, incomplete depigmentation, complete depigmentation, complete depigmentation plus partial leukotrichia, and complete depigmentation plus complete leukotrichia), and disease progression (spreading), scored −1 to +1 (ongoing subclinical repigmentation, similar limits, and ongoing subclinical depigmentation). A total score for the aforementioned parameters was calculated with values ranging from 0 to 16 for staging and −4 to +4 for spreading.

The following demographic and clinical information was taken into consideration: sex, age, age at onset of the disease, phototype, Koebner phenomenon, presence of Sutton naevi, personal history of premature hair graying (more than 50% of white hair before the age of 40 years), emotional stress as a triggering factor, previous episodes of repigmentation, inflammation/pruritus of the macules, and modality of onset of vitiligo.

We also evaluated personal and familial history of autoimmune diseases (pernicious anemia, Addison's disease, systemic lupus erythematosus, inflammatory bowel disease, alopecia areata, multiple sclerosis, myasthenia gravis, rheumatoid arthritis, scleroderma, Sjogren's syndrome, coeliac disease), thyroid disorders (in general and specifically autoimmune thyroid disorders (ATD) or nodules), cardiovascular, psychiatric, and neurological diseases, diabetes, hypertension, and dermatological diseases (in general and specifically psoriasis, atopic dermatitis, and melanoma). Familial history of vitiligo was also investigated.

Laboratory examinations included serum levels of triiodothyronine (T3) and thyroxine (T4) thyroid stimulating hormone (TSH) and the following autoantibody titers: anti-thyroglobulin (anti-TG), anti-thyroperoxidase (anti-TPO), anti-TSH receptor (anti-TSHR), anti-parietal cell antibody (APCA), anti-smooth muscle antibody (ASMA), anti-nuclear antibody (ANA), and extractable nuclear antibody (ENA).

### 2.2. Statistical Analysis

Characteristics of the study population were described using mean, standard deviations, frequencies, and percentages. In order to recognize factors associated with an age of onset >40 years and an age of onset <12 years, comparisons between groups were conducted by univariate and multivariate conditional logistic regression. Every possible potential predictor of the above-mentioned age of onset was firstly assessed individually, using the chi square/Fisher tests for categorical data and Student's *t*-test for quantitative data in a univariate analysis. The odds ratios (OR), the corresponding 95% confidence intervals (CIs), and *P* values were computed. Secondarily, every predictor was assessed using multivariate analysis, with odds ratios and associated 95% confidence intervals.

All *P* values < 0.05 were considered to be statistically significant. Statistical analyses were performed using the SPSS 15.0 for Windows program (Statistical Package for Social Science, SPSS Inc., Chicago, IL, USA).

## 3. Results

Patient demographics and clinical characteristics are shown in Table 1.

Results of the univariate analysis for patient characteristics in the adult onset group versus childhood onset disease revealed that phototype III (OR 0.49; *P* = 0.021), personal history of hypertension (OR 0.8; *P* < 0.001), head and neck involvement at disease onset (*P* = 0.007), and stress as an onset factor (OR 0.29; *P* < 0.001) were all positively associated with adult onset vitiligo. Moreover this group showed a significantly higher prevalence of thyroid disease (OR 0.27; *P* < 0.001), ATD (OR 0.22; *P* = 0.02), and thyroid nodules (OR 0.9; *P* = 0.002).

On the contrary, family history of dermatological conditions (OR 2.615; *P* = 0.002), family history of autoimmune diseases (OR 3.96; *P* = 0.020), phototype II (OR 2.02; *P* = 0.021), initial localization of the disease involving upper extremities (*P* = 0.009), and the presence of Koebner phenomenon (OR 3.00; *P* < 0.001) were all positively associated with childhood onset. Complete results of the univariate statistical analysis are listed in Table 1.

The results of the multivariate analysis pointed out that a family history of dermatological diseases (*P* = 0.003, OR 2.87) and Koebner phenomenon (*P* < 0.001, OR 4.73) were independently associated with an age of onset of vitiligo <12 years (Table 2). In contrast, stress at onset (*P* = 0.002, OR 0.34), personal history of thyroid diseases (*P* = 0.04, OR 0.4), ATD (*P* = 0.003, OR 0.23), and thyroid nodules (*P* = 0.001, OR 0.90) were independently associated with vitiligo age of onset >40 years.

In light of this data, we performed a further analysis in our patients affected by both vitiligo and thyroid disease, in order to evaluate whether or not the onset of vitiligo appeared prior to thyroid disease. According to our results, in the adult onset group, thyroid disease preceded vitiligo in a statistically significant number of patients (*P* = 0.014). However no statistical association was found when we performed this analysis considering ATD and thyroid nodules separately.

## 4. Discussion

Until now, there is a lack of information about the clinical and epidemiologic profile of adult onset vitiligo and only few studies have compared its characteristics with early onset disease [10–12]. The purpose of our study was to describe the effect of age at onset on disease features.

In our study, both sexes were equally affected and we did not find any significant difference in sex ratio (M : F, 1 : 1) between adult and childhood onset vitiligo. This result is in accordance with two previous papers [3, 13]. However, a female preponderance has been reported in childhood onset, compared with adult onset, vitiligo [4, 14]. Other studies [11, 12] comparing disease characteristics in childhood and later onset vitiligo (disease onset after the age of 12) reported a female preponderance in both groups.

The role of stress as an onset factor in vitiligo has been widely investigated, both in adults and in children [15, 16]. In our study patients with adult onset vitiligo recalled a stressful event with significantly higher frequency compared to those with childhood onset. This result is in agreement with the above-mentioned studies, regarding childhood and later onset vitiligo [11, 12]. However, according to Ezzedine et al. [11] patients with childhood onset vitiligo might be less able to recall such an event when seen first in adulthood, so a recall bias could be responsible for this difference.

Among the other significant results, we found a significantly higher prevalence of thyroid disease, ATD, and thyroid nodules in adult onset vitiligo patients. With regard to ATD, the reported diagnosis was confirmed by positive serum anti-TPO, anti-TG, anti-TSHR, and suggestive ultrasonography. The association between ATD and vitiligo has been widely explored in children [17]. On the contrary little is known about this relationship in adult onset vitiligo. As reported in a recent systemic review [18], patients with vitiligo show a 2.5-fold higher risk of developing ATD compared with patients without vitiligo. The risk of developing ATD seems to increase with age and this is in accordance with the assumption of the increase in prevalence of autoimmunity with age [19]. This could explain the statistical association assessed by our study, since mean age in adult onset group is higher than in the childhood onset one. Our results are also in agreement with the paper of Nicolaidou et al. [12], claiming that the risk of developing ATD was significantly lower when vitiligo appeared during childhood.

We also found an association between adult onset disease and thyroid nodules which, to our knowledge, has never been studied so far. This relationship could be due to the higher age of this group (56.3 yr versus 19.5 yr), which is considered an important risk factor for thyroid nodules [20].

Analyzing the age at onset of vitiligo compared with thyroid disease in patients affected by both diseases we found that, in the adult onset group, thyroid disease tends to precede vitiligo in a significant number of patients. According to recent studies [21, 22] ATD is associated with an increased systemic oxidative stress, which has been shown to play a major role in the pathogenesis of vitiligo [23]. In our analysis the relationship between adult onset vitiligo and previous thyroid disease was confirmed only when thyroid disease was considered on the whole, since statistical significance was lost when ATD and nodules were considered separately. However it should be considered that 68% of the cases of thyroid diseases we considered in our analysis were actually ATD, so it is possible that the loss of significance considering ATD alone could be related to the smaller number of patients.

Our analysis also revealed a significantly higher prevalence of Koebner phenomenon in the childhood onset group (*P* < 0.001), which had not been observed in other studies. A possible explanation for these figures could be that children are more likely to undergo skin traumas while playing and therefore to develop koebnerization of the skin. However our childhood onset group also included adults so other factors might be involved in this phenomenon.

Patients with childhood onset vitiligo were also more likely to have a positive history of dermatological diseases in their family, but when dermatological diseases were analyzed individually, no significant association between childhood vitiligo and a family history of vitiligo, melanoma, or psoriasis was found. Personal and familial history of atopic dermatitis were not associated with childhood onset vitiligo, in contrast with Ezzedine et al. [11]. This difference could be due to a recall error, since atopic dermatitis usually develops in the first year of life, and the majority of affected individuals have resolution of disease by adulthood [24].

The limitations of the study include possible recall bias, especially in patients whose disease started many years before presentation to our clinic.

## 5. Conclusions

In conclusion, upon multivariate conditional regression, our study shows that adult and childhood onset vitiligo have different clinical features. Childhood onset vitiligo is significantly associated with family history of dermatological conditions and Koebner phenomenon, whereas adult onset vitiligo is associated with stress as an onset factor, thyroid disease, ATD, and thyroid nodules. Those differences must be kept in due consideration in order to suggest appropriate screening programs and follow-up; thyroid screening in particular should be suggested to all patients with vitiligo, but its importance is even stronger in adult onset vitiligo, since the risk of developing thyroid disease is higher in these patients.

## Figures and Tables

**Table 1:** Patients characteristics and complete results of the univariate analysis.

Parameter (**∗**)	Age of onset <12 years	Age of onset >40 years	*P*	OR
Mean age at onset	7 years (range 0–12)	50.25 years (range 40–83)		

Mean age at time of consultation	19.5 years (range 3–69)	56.3 years (range 41–85)		

Mean duration of the disease	12.2 years (range 0.3–62)	6.2 years (range 0.2–30)		

Sex				
Female	34 (50.0%)	68 (55.2%)	0.438	1.236
Male	34 (50.0%)	55 (44.8%)		

Type				
Segmental	4 (5.9%)	4 (3.3%)	0.463	0.552
Nonsegmental	62 (91.2%)	116 (96.6%)	0.173	2.806
Unclassified	2 (2.9%)	0	0.130	0.355

Family history				
Vitiligo	22 (32.3%)	25 (24.3%)	0.247	0.670
Psoriasis	17 (25.0%)	15 (14.5%)	0.087	0.511
Melanoma	4 (5.8%)	6 (5.8%)	0.988	0.990
Dermatological	36 (52.9%)	37 (34.2%)	**0.002**	**2.615**
Thyroid disease	23 (33.8%)	29 (26.8%)	0.324	0.718
Cardiovascular disease	9 (13.2%)	10 (10.0%)	0.516	0.728
Type I diabetes	1 (1.4%)	5 (4.9%)	0.231	3.490
Type II diabetes	12 (17.6%)	16 (15.8%)	0.921	0.878
Autoimmune disease	8 (11.7%)	4 (4.0%)	**0.020**	**3.96**
Hypertension	14 (20.5%)	13 (12.8%)	0.179	0.570
Neurologic disease	0 (0.0%)	1 (1.0%)	0.406	0.590

Personal history of				
Thyroid disease	13 (19.1%)	40 (37.7%)	**<0.001**	**0.27**
ATD	7 (10.6%)	27 (25.0%)	**0.020**	**0.22**
Thyroid nodules	0%	13 (12.0%)	**0.002**	**0.90**
Cardiovascular disease	3 (4.4%)	5 (5.0%)	1.000	1.152
Autoimmune disease	3 (4.4%)	7 (7.0%)	0.742	1.649
Type I diabetes	0 (0.0%)	2 (2.0%)	0.515	0.590
Type II diabetes	0 (0.0%)	3 (3.0%)	0.273	0.588
Hypertension	0 (0.0%)	18 (18.1%)	**<0.001**	**0.8**
Psychiatric disease	0 (0.0%)	3 (3.0%)	0.273	0.588
Neurologic disease	1 (1.5%)	2 (2.0%)	1.000	1.367

Phototype				
I	1 (1.5%)	0 (0.0%)	0.364	0.360
II	36 (52.9%)	44 (36.9%)	**0.034**	**0.521**
III	28 (41.2%)	68 (62.4%)	**0.036**	**1.905**
IV	2 (2.9%)	7 (5.9%)	0.491	2.063
V	1 (1.5%)	0 (0.0%)	0.364	0.360

Previous episode of repigmentation				
Spontaneous	4 (5.9%)	8 (8.0%)	0.764	1.376
Drug induced	27 (39.7%)	33 (32.9%)	0.349	0.737
Photoexposition	9 (13.2%)	9 (9.0%)	0.372	0.641

Type of Onset				
Sudden	30 (44.1%)	57 (48.7%)	0.618	1.165
Progressive	35 (51.5%)	62 (52.1%)	0.934	1.026

Leucotrichia	16 (23.5%)	35 (36.0%)	0.086	1.835

Sutton	8 (11.7%)	7 (8.9%)	0.580	0.739

Koebner	42 (61.7%)	43 (43.8%)	**<0.001**	**0.484**

Stress as onset factor	29 (42.6%)	67 (65.0%)	**<0.001**	**2.503**

^*∗*^Continuous variables such as age, age of onset, and vitiligo duration are reported as mean ± SD and expressed in years. For every other parameter evaluated, we report the number and the percentage of patients positive or negative for that parameter.

**Table 2:** Significant results of the multivariate conditional logistic regression.

Parameter	*P*	OR
Family history of dermatological disease	0.003	2.87
Personal thyroid diseases	0.04	0.4
Koebner	<0.001	4.73
Stress as onset factor	0.002	0.34
Personal ATD	0.003	0.23
Personal thyroid nodules	0.001	0.90
